# Community dialogue to enhance understanding of beliefs, behaviours and barriers to care for people living with liver disease and HBV infection in KwaZulu Natal, South Africa

**DOI:** 10.1016/j.jve.2024.100378

**Published:** 2024-06-10

**Authors:** Busangani Ngwenya, Motswedi Anderson, Nondumiso Mpanza, Welcome Mbokazi, Luthando Zuma, Thandeka Khoza, Gloria Sukali, Elizabeth Waddilove, Marion Delphin, Collins Iwuji, Ngcebo Mhlongo, Nomathamsanqa Majozi, Janet Seeley, Janine Upton, Guy Harling, Philippa C. Matthews, Anita Edwards

**Affiliations:** a Africa Health Research Institute, KwaZulu-Natal, South Africa; b The Francis Crick Institute, 1 Midland Road, London, NW1 1AT, UK; c Botswana Harvard Health Institute Partnership, Gaborone, Botswana; d Division of Infection and Immunity, University College London, Gower Street, London, WC1E 6BT, UK; e Department of Global Health and Infection, Brighton and Sussex Medical School, University of Sussex, Brighton, UK; f Department of Global Health and Development, London School of Hygiene and Tropical Medicine, London, UK; g School of Nursing and Public Health, University of KwaZulu-Natal, Durban, South Africa; h University College London Institute for Global Health, Mortimer Market Centre, Capper Street, London, WC1E 6JB, UK; i MRC/Wits Rural Public Health & Health Transitions Research Unit (Agincourt), University of the Witwatersrand, Johannesburg, Gauteng, South Africa; j Department of Infectious Diseases, University College London Hospital, Euston Road, London, NW1 2BU, UK

**Keywords:** Hepatitis B virus, HIV, Community engagement, Health literacy, South Africa, Public engagement, Stigma, Liver disease

## Abstract

**Introduction:**

The World Health Organisation (WHO) has set targets for the elimination of Hepatitis B virus (HBV), which include preventing new infections and reducing deaths. We explored beliefs, behaviours and barriers to diagnosis, prevention and treatment for people living with HBV infection (PLWHB) and those with liver disease in a rural South African population in KwaZulu-Natal, to gather information to inform research and support the development of improved clinical and public health services.

**Methods:**

Using an interdisciplinary approach (combining public engagement, social science, clinical and laboratory team members) we conducted a community dialogue with members of the Africa Health Research Institute (AHRI) Community Advisory Board (CAB). Notes from the discussions were used to write up an account from which themes were identified during a team debrief session for data analysis.

**Results:**

There was a lack of knowledge and awareness of HBV infection and transmission and prevention amongst CAB members, also reported among community members and healthcare workers. The participants recognised liver disease symptoms. Perceived causes of liver disease reported by the CAB were alcohol and non-adherence to HIV treatment. Barriers to care included stigma, poverty, and delays in referrals for HBV diagnosis and management.

**Conclusion:**

Understanding barriers to care is important to shape future services for diagnosis, treatment and prevention of HBV and liver disease which are accessible, affordable and acceptable to the local population. Education, awareness and advocacy for improved liver health care pathways are required to make them effective for local communities.

## 1 Introduction

Hepatitis B virus (HBV) infection and the resulting liver disease are significant public health problems in many populations in Africa.^1^ Ambitious international goals have been established to eliminate the threat of viral hepatitis to public health by the year 2030.^2^ However, education and advocacy have been neglected, clinical services are typically poorly developed, and research fails to represent global populations in which prevalence of HBV infection is highest.^3^, ^4^, ^5^ In South Africa, over 1.9 million people are living with chronic HBV infection,^6^ despite the incorporation of HBV vaccination into the national infant Expanded Programme for Immunisation (EPI) since 1995.^7^ In response to the neglected areas of education and advocacy there has been a call for enhanced interdisciplinary action by researchers.^8^^,^^9^

The discipline of Public Engagement typically uses ‘community dialogue’ events as a forum to allow individuals in the community where research is being conducted to express their views, which provides an understanding of the community's experiences, needs and resources. Many disciplines in the Social Sciences use similar methodologies, such as focus group discussions and natural group discussions, to gather data on the knowledge, perceptions, and attitudes of a community to facilitate consultative and collaborative co-design and co-creation in the delivery of bespoke interventions that acknowledge the complexities of a community.

Using an inter-disciplinary approach, we sought to harness the strengths of these two approaches for enhanced engagement of the community as stakeholders and co-designers in ‘EVOLVE-HBV’, a translational research programme focusing on HBV infection, based at the Africa 10.13039/100005622Health Research Institute (10.13039/100016237AHRI) in South Africa, in a rural resource-constrained setting previously described.^10^ We set out to explore the current knowledge and beliefs, behaviours and barriers to diagnosis, prevention and treatment for people living with HBV infection (PLWHB) and those with liver disease in this population in KwaZulu-Natal, with the aim of gathering information to inform research on context-appropriate approaches for education and advocacy, and ultimately to support the development of improved clinical and public health services.

## 2 Methods

### 2.1 Interdisciplinary approach

The EVOLVE-HBV project convened interdisciplinary team meetings that included members of the Public Engagement (PE) team, social scientists, laboratory scientists, and clinicians at AHRI in KwaZulu-Natal. AHRI social scientists have extensive experience in conducting group discussions, and the PE team conduct regular community dialogue events within the research community. The interdisciplinary team shared and engaged in the methodologies used by the different disciplines and explored ways to best achieve the combined objectives of the activity and obtain good data. It was agreed that a topic guide would help to structure the community dialogue which typically would be a listening exercise and less structured than a Focus Group Discussion (topic guide available on-line^11^). The interdisciplinary team explored and planned the facilitation of the dialogue as well as the data collection and data analysis methods to be used. The clinical team and laboratory team had drafted an education pamphlet about the EVOLVE-HBV study, that would be used to provide information to potential research participants, and to enhance health literacy about liver disease and HBV. Plans for obtaining the community's views about the wording and the information on the pamphlet were discussed.

### 2.2 Participants

The PE team convened the Community Dialogue in January 2024 in a meeting room in the town within the research community where the EVOLVE-HBV study will take place, which is within the AHRI demographic surveillance area in northern KwaZulu-Natal.^10^ Participants were purposively recruited by inviting all members of the AHRI Community Advisory Board (CAB). The CAB members who responded to the invitation were 18 females and 9 males, aged 29–55, all of whom live in the rural communities of the AHRI demographic surveillance area and have been elected by the community to represent the research population as per the AHRI internal protocol.

### 2.3 Procedures

The PE team led the community dialogue using the topic guide to facilitate areas of discussion to be covered during the meeting. The social scientists contributed with probing questions in areas where more details about the responses were needed. Detailed notes, including observations of group dynamics and interactions, were recorded by both teams during the meeting. After brief introductions, initial discussion was held before CAB members were offered any information about HBV, so that their baseline knowledge, experience, and opinions could be heard. The local isiZulu language was used for communication, with a member of the PE team providing real-time translation into English for team members not fluent in isiZulu.

The clinical and laboratory study team then briefly presented information and pamphlets on liver disease and HBV infection. This included providing details of the EVOLVE-HBV research study,^11^ which aims to develop insights into HBV epidemiology, clinical disease burden and molecular characteristics in this population setting, and to provide an evidence-base that will support enhancements to care delivery and preventive interventions. Participants were then asked to arrange themselves into three smaller groups for further discussion of topics arising from the discussions and the presentation. In particular the smaller groups were asked to review and critique the wording and the information provided in the pamphlets to be used by the EVOLVE-HBV study.^11^

Refreshments were provided at the start and end of the Community Dialogue event. Reimbursement for time and travel was provided for CAB members, as per routine compensation protocol.^12^

### 2.4 Data analysis

Following the event, a debriefing meeting was held between the PE and social science staff who took part, and members of the clinical and laboratory team from the EVOLVE-HBV study to discuss the outcomes from the discussions. This provided ideas for additional themes, which augmented the topics agreed in advance of the event which guided the discussion. A list of codes was then derived from the combination of topics and the additional themes. Two team members, one from the social science team and one from PE reviewed the notes from the session and sorted the material using the list of codes. The principal investigator of the EVOLVE-HBV project, who attended the dialogue event, used their output as the basis for the first draft of the results section of this paper. This draft was discussed and edited by all co-authors to arrive at the account of the results from the discussion presented below.

### 2.5 Governance and ethics

This event was convened under the terms of the EVOLVE-HBV ethics, University of KwaZulu Natal (UKZN) Biomedical Research Ethics Committee (BREC) (ref. 00004495/2022) and University College London, UK ethics committee (ref. 23,221/001 EVOLVE-HBV). Participants were informed of the aims of the meeting in advance (at the time of invitation) and at the outset of the Community Dialogue event. We did not audio-record the discussions, but written notes were collected during the meeting (in anonymised format). We asked for consent to take photos to be shared to showcase the event and feedback to funders and the wider research community. We asked participants to engage in free speech to share their own views, while maintaining respect for others and ensuring all had the opportunity to contribute. We provided assurance that confidentiality would be respected such that outputs would be reported on behalf of the group and no individual participant would be identified in relation to their specific contributions, beliefs, experiences or feedback.

## 3 Results

### 3.1 Awareness and knowledge of liver disease and HBV

Participants recognised and shared information about liver disease, but had less awareness and knowledge about HBV infection. They observed that the signs of liver disease that are reported and recognised in this community include yellow hands and eyes, swollen ankles, and loss of appetite. There was also an observation that these signs typically reflect advanced or severe disease with poor outcomes, such that by the time someone is admitted to hospital the community expects them to die imminently.

Heavy alcohol use and lack of adherence to HIV treatment were raised as the primary causes of liver disease and were also suggested to be ‘causes of hepatitis B’. Although they generally recognised that HBV is present in their community, participants typically reported that they are not aware of personally knowing anyone living with HBV infection, and liver disease was not clearly linked to HBV infection in community narratives. Sexual exposure, tobacco smoking, and the use of snuff were also raised as possible causes of poor liver health. Participants reported assumptions that liver disease is a ‘white person's disease’ or a ‘rich disease’, such that local communities may not perceive HBV to be a relevant local health concern.

Terms used for liver disease in isiZulu are general and do not relate specifically to viral hepatitis infections. No specific local terms are recognised for hepatitis B infection and many contributors struggled to say the term “hepatitis B″ in English. Study team members raised concerns with the CAB members about the confusion between HBV and HPV (human papillomavirus), and between ‘hepatitis’ and ‘herpes’ virus infections due to similarities in nomenclature, which may not easily be discriminated. The study team also advised of the need to share information on other causes of viral hepatitis besides HBV such as hepatitis A, C, D and E viruses, as the differences are not clear to the public. Based on this feedback, additional details were incorporated in the information provided to the CAB, and in written materials for wider use in the community.

One perception that was raised in the discussions was the view that the cause of liver disease could be linked to local traditional medications such as ‘love potions’ which are used in settings where people practice polygamy. There were also concerns about the impact of prescribed medication on liver health, including antiretroviral therapy (ART) for HIV treatment. This perception stemmed from knowledge about regular liver function monitoring that may be recommended for those on ART. The case of an infant who had been diagnosed with liver disease was raised and discussed, with participants perplexed that this could not be due to alcohol or drug exposure but unaware of any other possible causes.

Despite the clinical team providing information about the advantages of ART in the prevention and treatment of HBV infection, no further discussion or awareness of the potential of HBV prevention methods such as testing, vaccination or antiviral prophylaxis was apparent from the participants. However, the need for increased health awareness was highlighted as a route to empowering individuals and communities to pursue improved health outcomes for people living with HBV, as well as to reduce HBV transmission through health education, disease literacy, reduced stigmatisation, and acceptance of HBV vaccination. Awareness of other endemic infections, such as tuberculosis (TB) and HIV, was observed by participants to be much better, as these conditions are perceived to be more common and have been prioritised by health messaging and research projects. Participants reported that awareness about HBV infection is also lacking among community care workers and healthcare providers and advocated for better training of these groups.

### 3.2 Impact of liver disease

People living with Hepatitis B (PLWHB) and those suffering from the consequences of liver disease in this community were reported to experience financial difficulties and a poor quality of life due to their lack of access to appropriate health care. Stigma and discrimination were mentioned as challenges. Participants described how symptoms of liver disease are immediately assumed to represent untreated HIV infection, which is then associated with stigma, and a loss of connections and withdrawal of support from families and communities. Care givers, family members or other members of the community can also experience discrimination if they try to support someone suffering from liver disease. If someone discloses that they have HBV infection, people think he/she is a witch or he/she is a victim of witchcraft and there is even a risk that they are subjected to personal threat as a result. Participants reported that ‘as a community we judge each other’ and ‘there is no support’. The participants noted that it would be difficult for an individual to know to whom they could disclose a diagnosis of chronic infection, given fears around discrimination and stigma.

### 3.3 Approaches to management and prevention of liver disease

Participants said that community members have pluralistic approaches to the treatment of liver disease, typically consulting traditional healers if there is limited access or assistance from medical practitioners. After having been provided with information on treatment for HBV infection, the participants reflected that the lack of cure strategies could be a potential barrier to engagement with HBV diagnosis or adherence to long term treatment. A potential collaboration between researchers and traditional healers to improve knowledge and awareness about liver disease and HBV amongst community members was highlighted as an area to be explored.

### 3.4 Access to healthcare

Poverty was perceived as being the primary barrier to access to healthcare. For those advised to take long-term treatment, adherence could be limited, as those without food security must prioritise feeding their family over treatment costs. Even if medication is provided free of charge, travel to health facilities and time away from other responsibilities imposes costs. In addition to the financial cost, the participants reported that transport is also slow, and a typical experience is that on arrival at a clinic there are long waiting times, leading many people to ‘give up’ and seek support from local traditional healers, who are accessible within their own communities.

Experiences of engagement with health services were reported to be intimidating or dismissive of people's understanding of their condition, with reports that people seeking help ‘feel ignored’ and healthcare practitioners thinking ‘they know best’, without being willing to listen. Delays in being referred for a liver disease diagnosis and slow turnaround times for laboratory tests were a concern, with reports that people in the community have died while awaiting results. Furthermore, absence of a specialist (Hepatologist) at the local district hospital may also contribute to an inadequate/slow referral system for HBV. There was a perception that there has been less focus on liver diseases than on other conditions. The discussion among participants focused on the need for development of equitable, friendly, person-centric services with the observation that with better support and education, health service utilisation would be more efficient.

The participants discussed whether the private sector needs to be more engaged, but generally felt this is not relevant in this community, in which private health care provision is not accessible or affordable.

### 3.5 Public health interventions and policy

The participants suggested the need for significant strengthening of health strategy and policy. Some participants were in favour of making HBV testing compulsory, or at least offering it as a routine standard alongside other available testing programmes (for example, diabetes screening). The idea of door-to-door testing (using point of care tests that can give quick results) was raised and supported by the participants, based on experience of such initiatives used for COVID-19 and HIV in the past. Although engagement with vaccine programmes has been variable, the participants said that community members are generally willing to accept vaccines as they are understood to prevent disease, particularly if given early in life.

### 3.6 Recommendations by participants

The CAB members recommended that education and information about health services and translational research for liver disease should be made more appealing and interesting, particularly to draw young people and enhance attendance by community members. Drama and sports events were suggested as opportunities for wider reach, potentially using branded materials and prizes as incentives for participation. The CAB recommended dissemination of information about HBV through posters in health facilities, social media (including community Facebook pages), local radio and pamphlets, and suggested that information should be made available at diverse sites not just in healthcare settings (churches, schools and clubs were suggested venues).

## 4 Discussion

While we found evidence that there is some knowledge on the causes, symptoms and consequences of liver disease, there is generally a low awareness of HBV, and the community members in this setting are heavily reliant on traditional medicine as a first-line intervention. Access to information, diagnosis, treatment, and follow-up is inhibited by barriers including poverty and inadequate access to health services. Stigma and discrimination have a significant negative effect on health seeking and quality of life, as previously documented for PLWHB.^13^ Language and the lack of terminology for HBV in isiZulu may be a further barrier to wider discussion and understanding.^14^

We found that our interdisciplinary approach offered an opportunity for the enhancement of understanding of HBV and liver disease amongst the CAB members at AHRI. This is in keeping with prior experience; for example, involvement of CAB members was reported to play a role in prevention of HIV and other sexually transmitted infections.^15^ The collaboration between the PE team and the social scientists was valuable in ensuring that the knowledge and expertise from the different fields is integrated to achieve the objectives of the EVOLVE-HBV study. Our experience with this event could provide an example for the further engagement of CAB members in research co-creation and implementation, resulting in longer term opportunities for education, community participation, and improved health literacy, in similar settings.

### 4.1 Limitations

While the CAB is selected to represent a cross-section of the community engaged in research in the AHRI demographic surveillance area, there are nevertheless potential limitations in the views expressed during community dialogues. All members of the CAB were invited to participate, but the group who arrived for the event were all unemployed and able to attend during the daytime in the week. By its nature, this group has been exposed to many health-related programmes, and is therefore likely to be more aware of health issues, and better able to communicate their knowledge compared to the general wider community.

It is possible that having mixed age and gender groups influenced discussion, with younger people, for example, being inhibited from sharing in the presence of their elders**.** These factors should be considered in future community dialogues. Further limitations to be addressed in future interdisciplinary events are the lack of objective scientific measures of success of the methods used. Further thought and development of such measures will improve the scientific rigour of both PE and social science methodologies. Finally, including science communication practitioners and health literacy experts in the team may further enhance the success of the interdisciplinary approach to co-creation of health interventions.

## 5 Conclusion

Our collaborative interdisciplinary approach can be used for future events to optimize partnerships and learning across different disciplines. Based on experiences reported and recommendations proposed by CAB members, it became clear that there is a need for new HBV care pathways. More resources are needed for the training of public engagement and research teams as well as community workers and healthcare providers in HBV and more general liver disease. Further HBV clinical research carries a promise of hope for improvement both in delivery of short-term and longer-term impact.

## Funding

This work was funded by 10.13039/501100000765University College London ‘Grand Challenges’ award for cross-disciplinary collaboration: ‘Beliefs, Behaviours and Barriers that influence access to hepatitis B interventions’ (Matthews and Harling), and through the 10.13039/100010438Francis Crick Institute core funding for the EVOLVE-HBV programme (Matthews, ref. CC2223). G.H. is supported by a fellowship from the 10.13039/100010269Wellcome Trust and 10.13039/501100000288Royal Society [grant number 210479/Z/18/Z]. 10.13039/100016237AHRI is core funded through the 10.13039/100010269Wellcome Trust [grant number 227167/Z/23/Z]. For the purpose of open access, the author has applied a CC BY public copyright licence to any Author Accepted Manuscript version arising from this submission.

## CRediT authorship contribution statement

**Busangani Ngwenya:** Formal analysis, Investigation, Methodology, Writing – original draft, Writing – review & editing, Data curation. **Motswedi Anderson:** Data curation, Formal analysis, Investigation, Methodology, Supervision, Writing – original draft, Writing – review & editing. **Nondumiso Mpanza:** Data curation, Formal analysis, Investigation, Methodology, Writing – original draft, Writing – review & editing. **Welcome Mbokazi:** Formal analysis, Investigation, Methodology, Writing – original draft, Writing – review & editing. **Luthando Zuma:** Data curation, Investigation, Methodology, Writing – review & editing. **Thandeka Khoza:** Methodology, Supervision, Writing – review & editing. **Gloria Sukali:** Data curation, Investigation, Writing – review & editing. **Elizabeth Waddilove:** Data curation, Investigation, Writing – review & editing. **Marion Delphin:** Data curation, Investigation, Writing – review & editing. **Collins Iwuji:** Conceptualization, Project administration, Supervision, Writing – review & editing. **Ngcebo Mhlongo:** Conceptualization, Project administration, Supervision, Writing – review & editing. **Nomathamsanqa Majozi:** Methodology, Supervision, Writing – review & editing. **Janet Seeley:** Conceptualization, Formal analysis, Methodology, Supervision, Validation, Writing – review & editing. **Janine Upton:** Investigation, Methodology, Supervision, Writing – review & editing. **Guy Harling:** Conceptualization, Formal analysis, Methodology, Supervision, Validation, Writing – review & editing. **Philippa C. Matthews:** Conceptualization, Data curation, Formal analysis, Funding acquisition, Investigation, Methodology, Project administration, Resources, Supervision, Validation, Writing – original draft, Writing – review & editing. **Anita Edwards:** Conceptualization, Data curation, Formal analysis, Investigation, Methodology, Resources, Supervision, Validation, Writing – original draft, Writing – review & editing.

## Declaration of competing interest

The authors declare that they have no known competing financial interests or personal relationships that could have influenced the work reported in this paper.

## Data Availability

The anonymised data are available upon reasonable request through the Africa Health Research Institute (AHRI) data repository at https://data.ahri.org/ index.php/home.
